# Passive exercise is an effective alternative to HRT for restoring OVX induced mitochondrial dysfunction in skeletal muscle

**DOI:** 10.3389/fendo.2024.1356312

**Published:** 2024-01-31

**Authors:** Yi Hu, Biqing Fang, Xu Tian, Haiwei Wang, Xiangyang Tian, Fangfang Yu, Tao Li, Zhijie Yang, Rengfei Shi

**Affiliations:** School of Exercise and Health, Shanghai University of Sport, Shanghai, China

**Keywords:** passive exercise, whole-body vibration training, hormone replacement therapy, 17β-estradiol replacement, muscle weakness, mitochondrial function, ovariectomized mice

## Abstract

**Background:**

Postmenopausal women are more prone to develop muscle weakness, which is strongly associated with impairment of mitochondrial function in skeletal muscle. This study aimed to examine the impact of a passive exercise modality, whole-body vibration training (WBVT), on muscle mitochondrial function in ovariectomized (OVX) mice, in comparison with 17β-estradiol (E_2_) replacement.

**Methods:**

Female C57BL/6J mice were assigned to four groups: sham operation control group (Sham), ovariectomized group (OVX), OVX with E_2_ supplement group (OVX+E), and OVX with WBVT group (OVX+W). The estrous cycle, body weight, body composition, and muscle strength of the mice were monitored after the operation. Serum E_2_ level was assessed by enzyme-linked immunosorbent assay (ELISA). The ATP levels were determined using a luciferase-catalyzed bioluminescence assay. The activity of mitochondrial respiration chain complexes was evaluated using high-resolution respirometry (O2K). Expression levels of oxidative phosphorylation (OXPHOS), peroxisome proliferator-activated receptor gamma coactivator 1 alpha (PGC-1α), and mitochondrial transcription factor A (TFAM) were detected using western blotting.

**Results:**

We observed decreased muscle strength and impaired mitochondrial function in the skeletal muscle of OVX mice. The vibration training alleviated these impairments as much as the E_2_ supplement. In addition, the vibration training was superior to the ovariectomy and the estradiol replacement regarding the protein expression of PGC-1α and TFAM.

**Conclusion:**

WBVT improves the OVX-induced decline in muscle strength and impairment of mitochondrial function in the skeletal muscle. This passive exercise strategy may be useful as an alternative to E_2_ replacement for preventing menopausal muscular weakness. Further studies are needed to understand the effects of WBVT on various physiological systems, and precautions should be taken when implementing it in patient treatment.

## 1 Introduction

Progressive muscle weakness occurs inevitably with aging. Compared with age-matched males, postmenopausal women are more prone to develop muscle weakness due to the loss of estrogen with ovarian failure (1, 2). Estrogen is a crucial regulatory hormone, which, aside from its role in reproduction, affects physiological functions in multiple organs and tissues, including bone, white-adipose tissues, brain, and skeletal muscle (3). Studies with ovariectomized (OVX) and estrogen receptor α (ERα) knockout mice have shown that loss of estrogen signaling resulted in decreased grip strength and endurance (4, 5). Estrogen actions on preserving muscle strength are associated with phosphorylation of the regulatory light chain(RCL), inhibition of myocyte apoptosis, and maintenance of satellite cell function, although its exact mechanisms remain to be elucidated (6–8).

Mitochondria, as the cellular power station, are critical for maintaining skeletal muscle homeostasis and function by supplying energy through OXPHOS. Additionally, they play vital roles in modulating reactive oxygen species (ROS), Ca^2+^ homeostasis, and cell death (9). A recent clinical study suggested that mitochondrial impairment is a hallmark of pre-frailty development and the onset of decline in muscle function among the elderly (10). Indeed, mitochondria are an important target of estrogen. Estrogen receptors (ERα and ERβ) have been reported to be localized in the mitochondria of the mouse C_2_C_12_ myoblast cells, suggesting a possible direct effect of estrogen on mitochondrial function in the skeletal muscle (11, 12). Previous studies in rodents have shown that OVX-induced dysfunction of skeletal muscle mitochondria, manifested by reduced ATP levels, impaired mitochondrial respiratory function, and altered membrane biophysical properties (13, 14). These studies also demonstrate that E_2_ therapy restored mitochondrial alterations induced by ovariectomy.

Hormone replacement therapy (HRT) — estrogen alone or a combination of estrogen and progesterone — is an effective therapy for relieving menopausal symptoms. Accumulating research supports the positive effects of estrogen-based hormone therapy on maintaining muscle mass and strength, as well as overall health (15). However, the benefits and risks of HRT remain controversial due to the possible risks of chronic diseases, such as cardiovascular disease, breast cancer, venous thromboembolism, stroke, and dementia (16–18). Individualized evaluation is crucial to meticulously balance potential benefits and risks. Hence, the quest for a relatively secure alternative becomes imperative, specifically targeting the maintenance of muscle health and the attenuation of muscle functional decline in postmenopausal women.

Physical exercise constitute effective preventive and therapeutic strategies capable of attenuating muscle health decline (19, 20). However, the likelihood of women participating in active physical activity decreases with advancing age (21, 22). Consequently, passive exercise is garnering growing attention as an alternative. This modality offers significant advantages, especially for individuals incapable or unwilling of undertaking active training. The benefits span both physical and cognitive domains, with advantages including a lower exercise threshold, reduced physical burden, and positive implications in rehabilitation (23–25). Notably, among diverse passive exercise methods, whole-body vibration training (WBVT) emerges as a standout approach, gaining recognition as an alternative exercise method (24). In our study, we chose WBVT as a passive intervention to investigate its impact on skeletal muscle function in OVX mice by assessing mitochondrial status.

Therefore, the primary purpose of this study was to examine the possible effects of WBVT on mitochondrial dysfunction in the skeletal muscle of OVX mice and compare it with the treatment of placing E_2_ extended-release tablets. We hypothesized that passive exercise would be an alternative mechanism to E_2_ replacement to improve OVX-induced mitochondrial dysfunction in skeletal muscle.

## 2 Methods

### 2.1 Animals models

**Figure 1:**
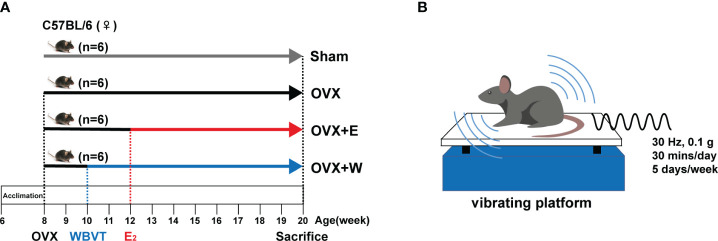
Schematic representation of the experimental design. **(A)** The schedule of E_2_ or WBVT treatment in OVX mice. **(B)** Schematic description of WBVT in mice.

The Experimental Animal Care and Use Committee of the Shanghai University of Sport approved animal experimentation. Six-week-old C57/BL6 female mice were purchased from the GemPharmatec Company (Nanjing, China). All mice were housed under the controlled temperature and lighting conditions of 22-25°C and a 12h light-dark cycle. As shown in **Figure 1A**, animals were randomly divided into four groups: sham operation control group (Sham); ovariectomized group (OVX); ovariectomized + E_2_ supplement group (OVX+E), and ovariectomized + WBVT group (OVX+W). Each cage accommodated six mice.

### 2.2 Surgical procedures: ovariectomy and sham operations

After two weeks of acclimation, female mice were randomly divided into two groups to receive a sham operation or bilateral ovariectomy, as described by our previous study (26). In brief, the mice were placed on the fixed table and were anesthetized with isoflurane. Ovariectomies were performed by cutting a 1cm incision on the back of the mouse and removing both ovaries. In sham surgery, only fat tissue of the same volume around the ovary was removed, and the wound was sutured. A successful ovariectomy was confirmed by microscopic examination of a vaginal smear for ten consecutive days.

### 2.3 Estradiol replacement

After a 4-week recovery period from ovariectomy, mice in group OVX+E received an E_2_-releasing pellet (SE-121, 0.36 mg, 60 days, IRA) implanted subcutaneously. Briefly, the mice were anesthetized with isoflurane, and the skin on the back was gently pulled up, a table-sized opening was cut, and inserted about 2 cm with tweezers to complete the implantation of sustained-release tablets. As previously reported, the dose of 17β-estradiol was chosen to obtain serum concentrations within the physiological range (27).

### 2.4 Whole body vibration training protocol

As shown in **Figure 1B**, mice in the OVX+W group were exposed daily to 30 min of vertical WBVT treatments (sine wave, 30 Hz, 0.1 g peak to peak) on a vibrating platform (LD-20BL, Longdate, Guangzhou, China). Vibration intervention took place from Monday to Friday, with Saturday and Sunday as the rest days. The training session started at 10:00 AM and lasted 10 weeks. The protocol used in this study was described in a previous study with a slight modification (28).

### 2.5 Grip test

Skeletal muscular strength in mice was quantified by the grip strength test (29). The limb grip strength of mice was measured by a Grip Strength Meter (YLS-13A, Jinan Yiyan Technology Co., Ltd. Jinan, China). After the mice grasped the sensor rod, the grip force peak in grams was automatically recorded. The average value of six measurements for each mouse was used for data analysis.

### 2.6 Determination of ATP content

The ATP content in skeletal muscle tissue was measured using an Enhanced ATP Assay Kit (Beyotime, China) according to the manufacturer’s instructions, and the results are shown in arbitrary units.

### 2.7 Mitochondrial respiration

Mitochondrial respiratory function in permeabilized myofiber bundles was performed as described previously (13). Briefly, a small portion of freshly dissected red gastrocnemius muscle tissue was placed in buffer X (2.77 mM CaK_2_EGTA, 7.23 mM K_2_EGTA, 5.77 mM Na_2_ATP, 6.56 mM MgCl_2_·6H_2_O, 20 mM Taurine, 15 mM Na_2_Phosphocreatine, 20 mM Imidazole, 0.5 mM Dithiothreitol, 50 mM K-MES, pH=7.1) containing 52.5 μg/mL saponin for separation and incubated at 4°C for 30 minutes. Permeabilized fiber bundles were transferred to buffer Z (0.5 mM EGTA, 3 mM MgCl_2_·6H_2_O, 60 mM K-lactobionate, 20 mM Taurine, 10 mM K_2_HPO_4_, 20 mM HEPES, 110 mM Sucrose, 1 g/L BSA, pH=7.1) at 4°C for 15 min. At the end of the incubation, quickly blot the fiber with filter paper, measure the wet weight, and put it back in buffer Z. Mitochondrial respiration was measured by high-resolution respirometry (O2K, OROBOROS Innsbruck, Austria). The chamber was hyperoxygenated to ~450 mM and started with the addition of L-Malic acid (1 mM) and L-Glutamic acid (Glu; 10 mM), followed by sequential additions of Adenosine 5′-diphosphate (5 mM), Cytochrome C (10 μM), Succinate (10 mM), Rotenone (0.5 μM), Antimycin A (2.5 μM), Ascorbate sodium salt (2 mM), and TMPD (0.5 mM). The respiration rate was normalized to the wet weight of permeabilized fibers.

### 2.8 Western blot analysis

Total protein from gastrocnemius tissue was extracted with the RIPA lysate buffer containing protease inhibitor. The protein concentration was measured by BCA assay following the manufacturer’s instructions. Sample proteins were separated by SDS-page using electrophoresis and then electro-transferred to polyvinylidene fluoride membranes. After blocking, the membranes were immunoblotted with total OXPHOS antibody (1:250; Abcam), PGC-1α (1:1000; CST), and TFAM (1:1000; CST). GAPDH (1:1000; CST) was used as the control of total protein expression. The appropriate HRP-conjugated secondary antibodies (1:1000; Beyotime) were used to combinate with primary antibodies and the proteins were visualized with enhanced chemiluminescence. Specific band intensities were quantified with the Image J program.

### 2.9 Statistical analysis

Statistical analysis was conducted using SPSS v.20 (IBM, Armonk, NY, USA), and all values are expressed as the mean ± standard deviation of the mean (SD). The Student’s t-test was used to compare two groups, while multiple groups were compared by one-way ANOVA with the LSD procedure for comparison of means. P<0.05 was considered statistically significant.

## 3 Results

### 3.1 Muscle weakness was improved by E_2_ or WBVT in OVX mice

**Figure 2:**
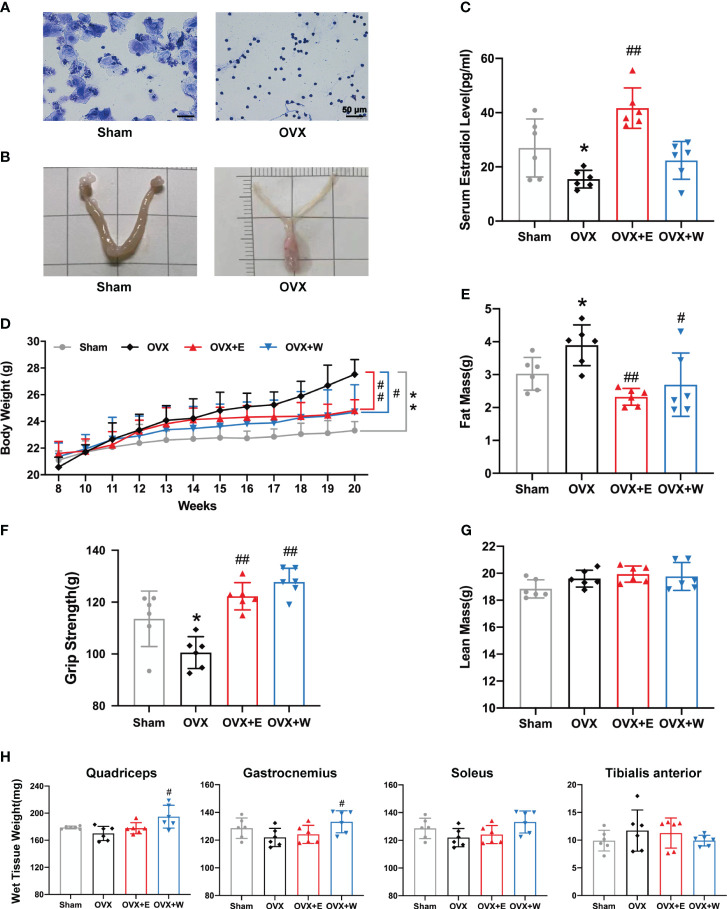
E_2_ or WBVT rescued muscle weakness in OVX mice. **(A)** Representative photomicrographs of vaginal cytology stained with crystal violet staining solution. Sham, estrus stage; OVX, diestrus stage. Scale bars, 50 μm. **(B)** Representative images of the uterus. **(C)** Serum estradiol level in each group at 20 weeks of age. **(D)** Changes in absolute body weight after surgery. **(E)** Fat mass. **(F)** Grip test. **(G)** Lean mass. **(H)** Wet tissue weight of hindlimb skeletal muscles. Values are means ± SD, *p < 0.05, **p < 0.01 versus Sham group and #p < 0.05, ##p < 0.01 versus OVX group(O), n = 6 mice/group.

To induce estrogen insufficiency, young C57BL/6 female mice were ovariectomized at eight weeks of age. We found that the OVX mice displayed a predominance of leukocytes, with minimal presence of keratinocytes, in vaginal secretions (**Figure 2A**). Moreover, uterine atrophy (**Figure 2B**) was observed, indicating changes in both the vaginal microenvironment and uterine morphology post-ovariectomy. We observed an apparent decrease in serum E_2_ level of OVX mice compared with Sham mice (**Figure 2C**), as the previous OVX mice model indicated (30, 31). Following ovariectomy, mice exhibited persistent weight gain (**Figure 2D**), and pre-euthanasia body composition measurements showed a significantly higher fat mass (**Figure 2E**) in ovariectomized mice than the sham-operated group. Collectively, these data confirmed that our ovariectomy-induced estrogen-deficient model was established successfully.

We investigated the impact of estrogen depletion on muscle function using a grip strength meter in mice. As show in **Figure 2F**, despite an increase in body weight, the limb strength of OVX mice showed an 11.5% reduction compared to the sham-operated animals. Previous studies have demonstrated that estrogen deficiency mediates decrements in muscle strength from both inadequate preservation of skeletal muscle mass and decrements in the quality of the remaining skeletal muscle (32). To assess if muscle weakness resulted from a loss of muscle mass, we measured the lean mass (**Figure 2G**) and wet weight of hind limb skeletal muscles (**Figure 2H**). Compared with the Sham group, no significant change was observed in lean mass. With regard to the wet weight of hind limb skeletal muscles, we found that the muscle mass of quadriceps (QUA), gastrocnemius (GAS), soleus (SOL), and tibialis anterior (TA) from the OVX group was similar to Sham group.

Two weeks after ovariectomy surgery, mice in the OVX+W group underwent a 10-week WBVT intervention. WBVT restored diminished grip strength in OVX mice (**Figure 2F**), despite no significant increase in serum estradiol concentration (**Figure 2C**). Eight weeks of E_2_ supplementation markedly elevated OVX mice’s serum estradiol concentration (**Figure 2C**), coupled with an enhancement in grip strength (**Figure 2F**). These results suggest that in reversing muscle weakness induced by estrogen deficiency, both WBVT and E_2_ supplementation exhibit similar effects, even though the former does not significantly elevate serum estradiol levels in OVX mice. With regard to lean mass, similar to OVX+E group, the OVX+W group had remained non-significantly altered when compared with the OVX mice (**Figure 2G**). Despite E_2_ supplementation, the mass of the four muscles remained unchanged compared to the OVX group. However, QUA and GAS muscle mass increased after WBVT (**Figure 2H**).

### 3.2 E_2_ or WBVT reverses the OVX-evoked mitochondrial dysfunction in the skeletal muscle

**Figure 3:**
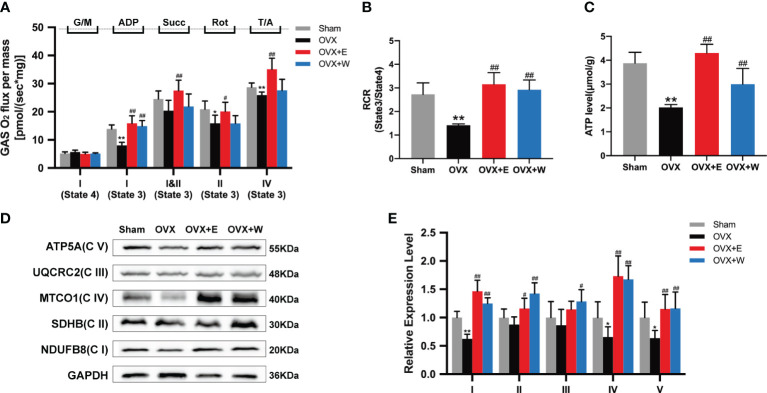
Effect of E_2_ or WBVT treated on mitochondrial respiratory function in OVX mice. **(A)** Respiration measured in permeabilized fiber bundles from red gastrocnemius. G/M, glutamate/malate; Succ, succinate; Rot, rotenone; AmA, antimycin A; T/A, TMPD (N,N,N’,N’-tetramethyl-p-phenylenediamine dihydrochloride)/ascorbic. **(B)** Mitochondrial respiratory control ratio (RCR). **(C)** Mitochondrial ATP content in skeletal muscle of each group. **(D)** Representative images of OXPHOS complexes subunits. **(E)** Differences in the OXPHOS complexes expression. Values are means ± SD, *p < 0.05, **p < 0.01 versus Sham group and #p < 0.05, ##p < 0.01 versus OVX group(O), n = 3~6 mice/group.

Given that mitochondrial respiratory function in skeletal muscle is affected by ovariectomy (33), permeabilized fiber bundles from the red portion of the GAS were prepared for high-resolution respirometry. As shown in **Figure 3A**, when compared with the Sham group, the rate of state 3 C I -linked respiration (+ADP) was significantly decreased in the OVX mice; whereas those were obviously increased in the OVX+E group or OVX+W group when compared with the OVX group. Moreover, we also found a decrease in the rate of state 3 C I+II-linked respiration (+succinate), state 3 C II-linked respiration (+rotenone), and state 3 C IV maximal respiration of the OVX mice compared to Sham group. Changes in these indices were significantly reversed after E_2_ supplementation. The rate of state 4 Complex (C) I-linked (glutamate/malate, no ADP) respiration was not changed among the groups. Dividing state 3 CI-linked respiration (+ADP) and state 4 CI-linked respiration (glutamate/malate, no ADP), we obtained the respiratory control ratio (RCR). As shown in **Figure 3B**, the RCR values significantly decreased post-ovariectomy. When compared to the OVX group, both the OVX+E and OVX+W groups exhibited a significant increase in RCR values.

In addition, we investigated the ATP production in the GAS muscle of the mice. The results showed that ATP production in the GAS muscle of OVX mice was significantly lower than that of the Sham group, OVX+E group, and OVX+W group (**Figure 3C**), indicating that estrogen supplementation or WBVT treated improved ATP production of skeletal muscle.

We next evaluated the OXPHOS protein expression in GAS muscle among the four groups. Similar to the decline in respiratory function, mitochondrial complexes I (NDUFB8), IV (MTCO1), and V (ATP5A) showed reduced expression in the OVX mice compared to the Sham group. However, no differences were observed for other oxidative phosphorylation (OXPHOS) subunits (**Figures 3D, E**). E_2_ supplement or WBVT treatment increased C I(NDUFB8), II(SDHB), IV(MTCO1) and V(ATP5A) in OVX mice (**Figures 3D, E**). Intervention with WBVT significantly increased C III(UQCRC2) expression, whereas the E_2_ supplement did not promote it as much as the WBVT. These results suggest that estrogen deficiency induces mitochondrial dysfunction in the skeletal muscle of mice, which can be alleviated by E_2_ supplementation or WBVT.

### 3.3 E_2_ or vibration training restores OVX-induced impairment of mitochondrial biogenesis in the skeletal muscle

**Figure 4:**
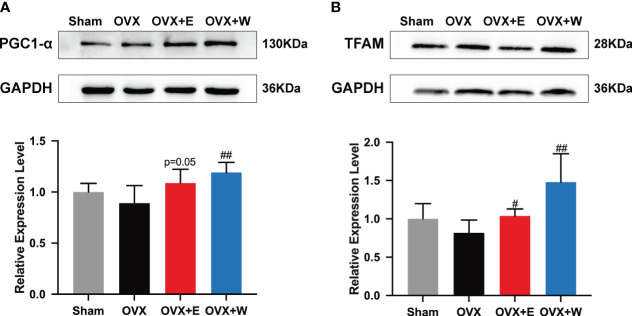
Effects of E_2_ or WBVT treated on mitochondrial biogenesis signaling in OVX mice. **(A)** PGC-1α. **(B)** TFAM. Values are means ± SD, #p < 0.05, ##p < 0.01 versus OVX group(O), n = 6 mice/group.

To investigate the effects of E_2_ supplementation or WBVT on mitochondrial biogenesis, protein expression levels of PGC-1α and TFAM in gastrocnemius muscle were measured (**Figure 4**). Although the expression of PGC-1α in gastrocnemius muscle of ovariectomized mice tended to decrease compared to sham group, the difference was not statistically significant. When compared with the OVX group, an increasing trend was observed for the OVX+E group. In contrast, WBVT significantly increased the expression of PGC-1α in the skeletal muscle. Regarding the expression of TFAM, ovariectomy did not alter it, whereas E_2_ or WBVT treatment increased it. The results suggested that WBVT can enhance the expression of PGC-1α and TFAM, both of which are related to mitochondrial biogenesis.

## 4 Discussion

In postmenopausal women, the decline in estrogen levels is implicated in the development of skeletal muscle strength loss, potentially predisposing individuals to compromised muscle health (34). Previous investigation identifies E_2_ deficiency as a potential catalyst for mitochondrial dysfunction (13), a factor that may play a role in the initiation of muscle weakness. Our research findings indicate that estrogen therapy has the capacity to counteract the detrimental effects associated with estrogen decline, offering a means to mitigate muscle weakness. Additionally, WBVT, a form of passive exercise, emerges as a viable alternative to estrogen therapy. It exhibits the ability to not only restore muscle weakness and mitochondrial dysfunction resulting from reduced estrogen levels but also to augment mitochondrial biogenesis.

Skeletal muscle weakness is considered a significant component of the loss of health-related fitness in postmenopausal females (34, 35). The induction of estrogen deficiency in rodents by ovariectomy is a common model of human menopause and is useful towards understanding estrogenic effects on muscle strength (36). Kitajima et al. (37) has demonstrated in their study that 24 weeks after ovariectomy, mice exhibited a reduction in muscle force generation and a significant decrease in the cross-sectional area (CSA) of the TA muscle compared to the control mice. In the current research, we noted an important reduction in muscle strength 12 weeks post-ovariectomy surgery, with no significant decrease in muscle mass. The duration of low estrogen levels may have varying effects on muscle mass, and indeed, the decline in strength with aging occurs at a greater rate than the decrease in muscle mass (36). Moreover, the present study found that an 8-weeks E_2_ replacement rescued ovariectomy-induced skeletal muscle weakness. Notably, as a form of passive exercise, WBVT intervention in OVX mice was also observed to alleviate muscle weakness caused by reduced estrogen levels, and the reversal effect was found to be superior to estrogen supplementation.

Reduced muscle function is linked to mitochondrial dysfunction (38, 39). Mitochondria are critical organelles in skeletal muscle responsible for regulating its metabolic status, including chemical energy (ATP) production, regulation of intracellular Ca^2+^ homeostasis, modulation of cell proliferation, and integration of apoptotic signaling (40, 41). In the present study, the loss of Ovarian E_2_ resulted in diminished ATP production, and WBVT treatment rescued this decline, similar to E_2_ therapy. The activity of mitochondrial complexes is directly associated with ATP generation. Multiple beneficial effects of E_2_ are reported to be mediated through improved CI activity (42). Our high-resolution respirometry results demonstrated that the rate of state 3 CI -linked respiration (+ADP) was reduced after OVX and restored by E_2_ supplementary. The WBVT group also observed alterations in mitochondrial respiratory state 3 (ADP coupling). What’s more, following WBVT or E_2_ supplementation, the reduced RCR values in OVX mice were increased, suggesting that both interventions can mitigate the decreased OXPHOS coupling efficiency caused by ovariectomy. Cavalcanti-de-Albuquerque et al. showed that ovariectomy resulted in reduced mitochondrial respiration capacity and a lower ATP synthase respiration rate, possibly due to a decrease in mitochondrial number (14). In our study, after 12 weeks of the ovariectomy, we observed a downregulation in the expression of the mitochondrial respiratory complexes, indicating that alterations in mitochondrial function may be attributed to the content of mitochondrial complexes. Previous studies have demonstrated that estrogen’s effects on mitochondrial function over a 2-week duration were not linked to changes in mitochondrial content (13), whereas prolonged estrogen deficiency for 15 weeks led to alterations in OXPHOS enzyme levels in OVX mice (4). E_2_ supplementation or WBVT can both increase the protein expression of mitochondrial complexes, reversing the decline of mitochondrial complexes content induced by estrogen deficiency.

Mitochondrial biogenesis stands as a pivotal component of mitochondrial quality control, exerting a significant influence on mitochondrial function (43, 44). Ren et al. (45) reported vibration intervention can enhances muscle strength and muscle mitochondrial biogenesis-related gene relative mRNA expression. PGC-1α, a master regulator of mitochondrial biogenesis, coordinates transcription to enhance mitochondrial mass and support tissue adaptation to increased energetic demands, concurrently upregulating TFAM essential for mitochondrial DNA functions (46, 47). Previous studies have shown that estrogen deficiency can reduce the expression levels of PGC-1α, influencing mitochondrial biogenesis (14, 36). Additionally, a whole-body vibration exercise program, incorporating resistance exercise and sustained vascular occlusion, increased the abundances of PGC-1α mRNA in skeletal muscle, indicating potential benefits of vibration exercise in mitochondrial biogenesis (48). Our research findings indicate that after 10 weeks of WBVT, the expression of PGC-1α and TFAM was upgraded in the skeletal muscle of the OVX mice, suggesting that vibration training has beneficial effects on mitochondrial biogenesis in the context of low estrogen level.

The dynamic balance of cellular mitochondrial content is maintained through the opposing processes of mitochondrial biogenesis and mitophagy (41). The main mitophagy pathway that has been investigated in the context of exercise involves PINK1 and Parkin (49). Currently, the impact of low estrogen levels or vibration training on mitophagy remains insufficiently examined. In our investigation, we specifically concentrated on proteins associated with mitochondrial biogenesis. Further studies are needed to elucidate the specific pathways by which WBVT affects mitochondrial function in the setting of estrogen deficiency, including changes in mitophagy-related proteins.

In our study, there was no statistical difference in serum estradiol levels between the OVX+W group and the OVX group. There is still controversy about whether exercise can change serum estradiol levels. Some studies have found that exercise intervention does improve the level of circulating estrogen in postmenopausal women (50), whereas others report divergent outcomes (51). In postmenopausal women, estrogen derives primarily from non-gonadal tissues, including adipose tissue, kidneys, brain, and skeletal muscle (52–54). Exercise have complex effects on these tissues, which may affect the synthesis and secretion of estrogen (55–57). In our experiment, the vibration group exhibited an upward trend in serum estradiol levels. This observation suggests a potential link between vibration training and its impact on adipose tissue, skeletal muscle, and other tissues. Further investigations are imperative to elucidate the intricate correlation between serum estrogen levels and various forms of exercise.

## 5 Conclusions

In summary, the present study demonstrates that WBVT, as a form of passive exercise, can ameliorate muscle weakness attributed to estrogen deficiency by mitigating mitochondrial dysfunction. Our proposed passive exercise strategy to alleviate the repercussions of mitochondrial impairment relies on the modulation of mitochondrial biogenesis protein expression. These discoveries substantiate the potential of passive exercise as a promising alternative therapeutic option to conventional estrogen supplementation. However, until how WBVT affects various physiological systems is fully understood, caution should be exercised when utilizing WBVT as a therapy for postmenopausal women and as a potential method for enhancing muscle strength.

## Data availability statement

The original contributions presented in the study are included in the article/supplementary material. Further inquiries can be directed to the corresponding authors.

## Ethics statement

The animal study was approved by The Experimental Animal Care and Use Committee of the Shanghai University of Sport. The study was conducted in accordance with the local legislation and institutional requirements.

## Author contributions

YH: Conceptualization, Data curation, Formal analysis, Resources, Writing – original draft, Writing – review & editing. BF: Conceptualization, Data curation, Formal analysis, Resources, Writing – original draft, Writing – review & editing. XuT: Conceptualization, Data curation, Formal analysis, Resources, Writing – original draft, Writing – review & editing. HW: Methodology, Software, Validation, Writing – review & editing. XYT: Methodology, Software, Validation, Writing – review & editing. FY: Methodology, Software, Validation, Writing – review & editing. TL: Methodology, Software, Validation, Writing – review & editing. ZY: Funding acquisition, Project administration, Supervision, Writing – original draft, Writing – review & editing. RS: Funding acquisition, Project administration, Supervision, Writing – original draft, Writing – review & editing.
